# Parental Depression in Childhood and Depressive Symptoms in Later Life

**DOI:** 10.1093/geroni/igaf122.4168

**Published:** 2025-12-31

**Authors:** Wenxing Wei, Aloen Townsend

**Affiliations:** Case Western Reserve University, Cleveland, Ohio, United States; Case Western Reserve University, Cleveland, Ohio, United States

## Abstract

There is limited research examining parental depression in childhood as a separate risk factor, rather than combining it with other adverse childhood experiences (ACEs). Based on life course theory, this study investigates the association between parental depression during respondents’ childhood (neither parent, father only, mother only, or both parents depressed) and later-life depressive symptoms and whether this association differs by respondents’ gender, while controlling for other ACEs, sociodemographic characteristics, and health. The study was based on a nationally representative sample of 4,635 males and 5,457 females aged 45 and older from the China Health and Retirement Longitudinal Study (CHARLS). ACE variables in the 2014 Life History Survey were merged with the harmonized 2018 CHARLS dataset. Multiple imputation addressed missing data. One-way ANOVA and hierarchical OLS multiple regression were conducted. The regression model explained significant variance (R2 = .229, p < .001). Both mother only depressed and both parents depressed were significantly associated with higher later-life depressive symptoms, compared to having neither parent depressed. Respondents with both parents depressed reported the highest depressive symptoms (b = 1.642, p < .001). Gender did not significantly moderate these relationships. The findings suggest that maternal depression and having both parents depressed have particularly negative long-term effects on respondents’ mental health, which should be considered in the development of protective strategies and policies. Even in middle and older age, the study findings highlight the importance of assessing family history of depression.

